# Characterization of the ligand binding pocket of the virulence regulator Rns, a member of the AraC/XylS family of transcription factors

**DOI:** 10.1128/msphere.00115-25

**Published:** 2025-07-24

**Authors:** Jessica D. Tolbert, Kacey M. Talbot, Christopher M. Bollinger, F. Jon Kull, George P. Munson, Charles R. Midgett

**Affiliations:** 1 Department of Chemistry, Dartmouth College 3728, Hanover, New Hampshire, USA; 2 Department of Microbiology & Immunology, University of Miami, Leonard M. Miller School of Medicine 12235, Miami, Florida, USA; Vanderbilt University Medical Center, Nashville, Tennessee, USA

**Keywords:** ETEC, fatty acid, decanoic acid, *E. coil*, virulence, inhibition

## Abstract

Diarrheal disease caused by Gram-negative enteric pathogens, such as enterotoxigenic *Escherichia coli* (ETEC), *Vibrio cholerae*, *Shigella* spp., and *Salmonella* spp., is a leading cause of morbidity and mortality of children, especially in low resource nations. While progress has been made in reducing this burden, there remains a need to develop effective therapies. Recently, we determined the structure of Rns, a member of the AraC/XylS family that regulates the expression of pili and other virulence factors in ETEC. The structure revealed decanoic acid bound between the N- and C-terminal domains. To test the hypothesis that bound decanoic acid directly inhibits Rns, we identified amino acid side chains predicted to be necessary for ligand binding. Removal of the positive side chains of R75 and H20 rendered Rns insensitive to fatty acid inhibition. Additionally, mutations designed to block decanoic acid binding also produced a variant Rns that was fatty acid insensitive. We also observed that this variant is structurally more flexible than wildtype Rns bound to decanoic acid, suggesting that fatty acid binding contributes to structural rigidity. These studies demonstrate that Rns binding pocket residues are critical for binding fatty acids, which result in inhibition of DNA binding and support our hypothesis that fatty acids must bind in the binding pocket to inhibit other AraC regulators. Further work by us and others suggests that inhibition of AraC virulence regulators by fatty acids is a common paradigm among many bacterial pathogens. Therefore, understanding the molecular basis of inhibition lays the groundwork for the development of small molecule therapeutics targeting enteric disease.

**IMPORTANCE:**

As antimicrobial resistance increases, it is critical to develop new strategies to combat these infections. One area of concern is bacteria that cause intestinal disease such as *Salmonella* species, *Vibrio cholerae*, *Shigella* species, and enterotoxigenic *Escherichia coli* (ETEC). ETEC is a leading cause of travelers’ diarrheal disease and a leading cause of mortality for children under 5 years old. To cause disease, ETEC requires the gene regulator Rns. Our previous work found that Rns was inhibited by a fatty acid. Here, we identify key features in the protein that are required for not only binding fatty acids but also for responding to them. This was done through a combination of microbiological as well as structural techniques of altered Rns proteins that can no longer bind fatty acid. Understanding how Rns is inhibited will lead to new ideas about how to target this class of proteins without causing antimicrobial resistance.

## INTRODUCTION

Diarrheal disease is the second leading cause of death in children worldwide, leading to an estimated half a million deaths annually (1). Enterotoxigenic *E. coli* (ETEC) accounts for close to 50,000 deaths of children under 5 years old (1). Survivors of repeated ETEC infections may suffer malnutrition during their critical developmental years and suffer comorbidities, such as stunting and long-term cognitive impairment (2, 3). Much effort has been expended to develop a vaccine against ETEC. However, vaccine development has mainly focused on pilins, and those efforts have been stymied by the antigenic diversity of pilins and suboptimal long-term immunity (4). This, combined with a rise in multidrug-resistant *E. coli* strains, highlights the need for alternative therapeutic solutions (5–7). One such approach is to focus on pathways specific to the pathogenic process, which should decrease the selection for antibiotic resistance (8).

ETEC and other enteric pathogens possess transcriptional regulators belonging to the AraC/XylS family that directly regulates the expression of genes involved in pathogenesis, including attachment factors, diarrheal-causing toxins, and other virulence factors, such as CexE, AcfA, and AcfD (9–13). Like other canonical AraC/XylS family members, these virulence regulators are defined by a structurally conserved DNA binding domain consisting of two helix-turn-helix motifs (14) and an amino-terminal domain that participates in dimerization and/or ligand interactions (15). Rns, a canonical AraC/XylS family member from ETEC, positively regulates its own expression and activates the expression of adhesive pili (11, 16–20). Without the expression of these pili, adherence to the host epithelium is impaired, and diarrheal disease is attenuated (21). Moreover, Rns is an attractive therapeutic target because it is well conserved across ETEC strains even though the pilins that it regulates are not. Rns also activates the expression of CexE, an outer membrane lipoprotein and virulence factor, as well as its secretion system (10, 22–24). It also represses the expression of *nlpA*, a gene that has been implicated in the biogenesis of outer membrane vesicles (12, 25). Thus, a clear understanding of Rns regulation could lead to new strategies to combat ETEC infections.

Until recently, there was no evidence that Rns was directly modulated by effectors, despite the known exogenous ligand regulation of many other AraC/XylS members (26–28). Previous work with other AraC members like ToxT in *V. cholerae* and work from other groups on regulators from *Salmonella* (HilD, HilC, RtsA) and *Shigella flexneri* VirF revealed the inhibitory effects of a range of long-chain fatty acids on regulator activity (29–34). This, combined with our work on Rns, has challenged the paradigm that the ability of AraC/XylS virulence regulators to bind DNA is regulated only by protein-protein interactions, showing DNA binding is also regulated by small molecules (35, 36).

**Fig 1:**
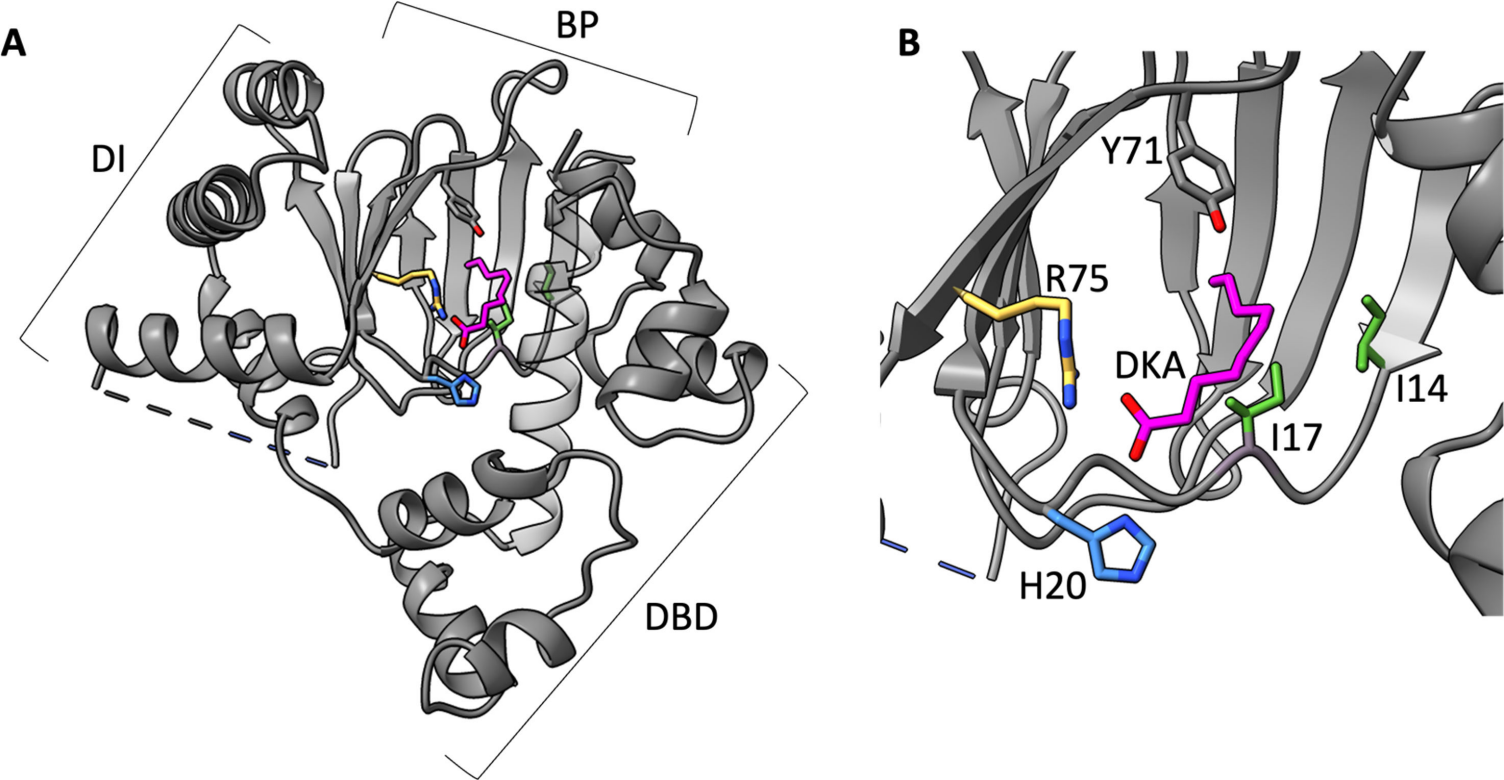
The structure of Rns with bound fatty acid. (**A**) Rns (PDB ID: 6XIU) bound to saturated, decanoic acid (DKA). Depicted are the three domains: dimerization interface (DI), binding pocket (BP), and DNA binding domain (DBD). (**B**) Close up of the pocket showing R75 in yellow, H20 in blue, I14 and I17 in green, and Y71 in gray. Residue Y71 is used as a reference for all structures as an unvarying pocket element.

The first crystal structure of Rns revealed a binding pocket between the N- and C-terminal domains occupied by the 10-carbon saturated fatty acid, decanoic acid (36) (Fig. 1). To investigate the biological significance of our crystallographic discovery, we cultured ETEC strains with decanoic acid concentrations from 20 µM to 1.2 mM (36). Those experiments revealed that the expression of pilins and CexE decreased with increasing concentrations of decanoic acid and that expression of pilins was undetectable in the presence of ~300 µM decanoic acid. We further showed that decanoic acid inhibited the expression of β-galactosidase in a Lac reporter strain in which the Rns-dependent CS3 pilin promoter (CS3p) drives the expression of *lacZ*. These effects were specific for proteins within the Rns regulon because exogenous decanoic acid had negligible effect on the expression of Rns-independent proteins such as flagellin. Although these results did not reveal a molecular mechanism, they suggest that the activity of Rns is abolished when decanoic acid occupies its binding pocket.

In this study, we identify amino acid side chains in the Rns binding pocket that are critical for both fatty acid binding and inhibition of Rns. Specifically, we focused on the role of residues that stabilize the negative charge of the fatty acid carboxylate head group, as well as those positioned to block fatty acid binding following the introduction of bulkier side chains. Altering the electrostatic environment and size of the pocket decreased the response of Rns to fatty acid, supporting our hypothesis that direct binding of fatty acid to the Rns pocket is critical for its inhibitory response. These results provide evidence that AraC family virulence regulators share a common inhibitory mechanism, laying the groundwork for future development of antivirulence therapeutics against a wide array of enteric pathogens.

## RESULTS

### Decanoic acid interferes with Rns DNA binding

**Fig 2:**
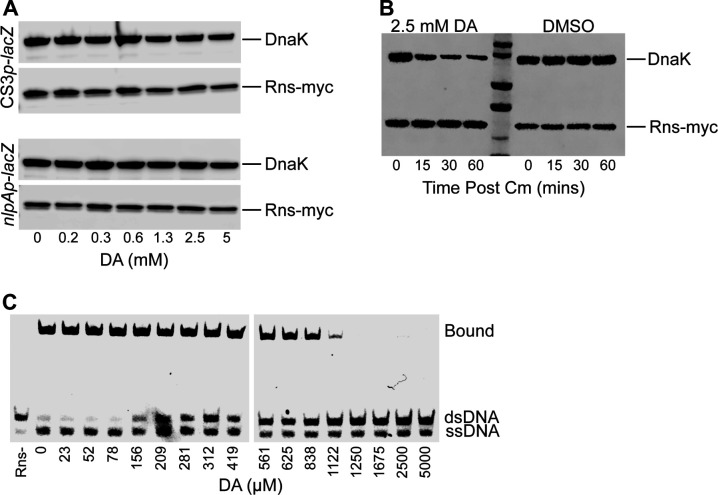
Decanoic acid does not destabilize Rns but does abolish its ability to bind DNA. (**A**) Western blots of whole cell lysates of K-12 Lac reporter strains GPM1072 and GPM1080 transformed with pGPMRns-myc. Rns-myc expression is persistent *in situ* relative to the DnaK loading control even at concentrations of decanoic acid that abolish its inhibitory activity. (**B**) Western blot of plasmid-expressed Rns-myc in ETEC strain H10407 *rns::ka*n (GPM1236) in which translation was inhibited with 30 µg/mL chloramphenicol (Cm). The apparent turnover rate of Rns was observed to be comparable in the presence or absence of decanoic acid. (**C**) EMSA of MBP-Rns with DNA containing a prototypical Rns binding site described in Materials and Methods. Decanoic acid abolishes the ability of Rns to bind the DNA duplex in a dose-dependent manner.

As discussed above, we have previously established that Rns binds decanoic acid and that decanoic acid inhibits its activity as a transcriptional regulator (36). To elucidate the molecular mechanism(s) of the latter observation, we considered the possibility that decanoic acid binding increases the turnover/degradation of Rns *in situ*. As there are no validated antibodies for the detection of native Rns, we evaluated decanoic acid-dependent turnover of epitope-tagged Rns in K-12 Lac reporter strains (Fig. 2A). Although we have previously shown that 1–3 mM decanoic acid is sufficient to inhibit Rns activity in these strains, we did not observe a dramatic decrease in the level of Rns even at 5 mM decanoic acid. Likewise, a time course in which *de novo* translation was inhibited in an ETEC strain found little difference between decanoic acid-treated and DMSO control cultures (Fig. 2B). Although qualitative, these results suggest decanoic acid binding to Rns does not appreciably affect its stability *in situ*.

As a transcription factor, the activity of Rns is dependent upon its ability to bind DNA. Thus, in the absence of apparent decanoic acid-dependent turnover of Rns, we considered the possibility that fatty acid binding to the pocket abolishes DNA binding. For *in vitro* DNA binding studies, we used an MBP-Rns fusion to increase the solubility of Rns (37). Low solubility is a well-known characteristic of AraC family members, and it has previously been shown that Rns precipitates after cleavage from MBP while the latter remains in solution (37). Despite its bulk, the MBP solubility tag does not interfere with the ability of Rns to activate Rns-dependent promoters *in situ* (38). Like Rns, the MBP-Rns fusion is also inhibited by decanoic acid *in situ* (Fig. S1). Consistent with our *in situ* observations, *in vitro* experiments revealed that decanoic acid abolished the ability of MBP-Rns to bind a DNA duplex carrying a consensus Rns binding site (GTGTTATTTTTTTATC) derived from the alignment of over 30 experimentally defined sites (Fig. 2C) (12, 16, 19, 20, 38–41; G. P. Munson, unpublished data). These results suggest that the inhibitory effects of decanoic acid *in situ* are likely produced by its ability to abolish the DNA binding of the transcription factor.

### Electrostatic interactions contribute to occupancy of the decanoic acid binding pocket

**Fig 3:**
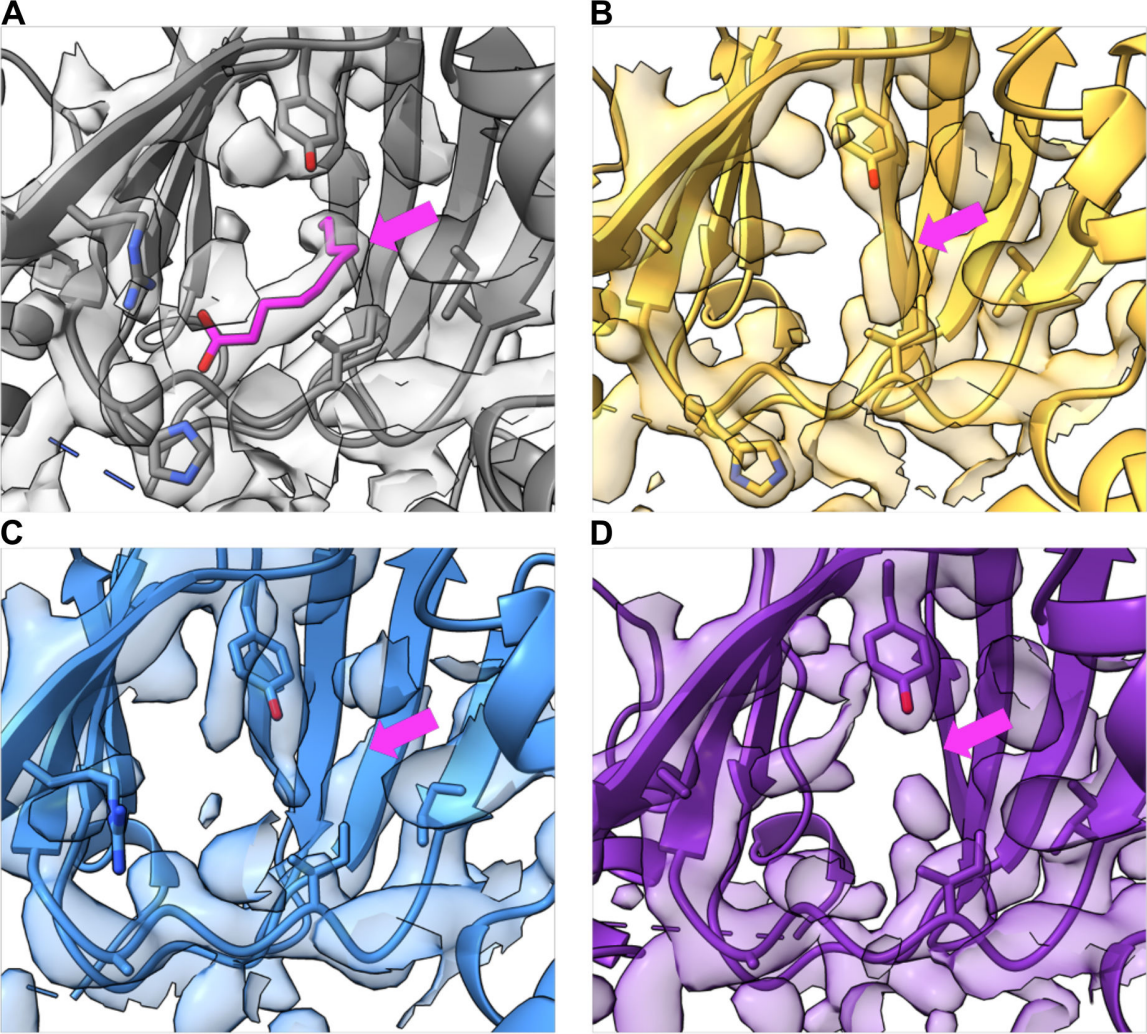
Rns pocket mutants abolish previously observed electrostatic interaction potential. (**A**) WT Rns (gray) with bound decanoic acid. Variants (**B**) R75A (yellow, PDB ID: 8VRQ), (**C**) H20A (blue, PDB ID: 9CA5), and (**D**) H20A/R75A (purple, PDB ID: 8VST) do not contain electron density consistent with bound ligand. Y71 is visible in all the structures. In panel **A**, the pink arrow points to the decanoic acid in the wild-type structure. In panels **B–D**, the pink arrow points to the area where decanoic acid is bound in the WT structure.

Our previous work with Rns-decanoic acid cocrystals showed the positively charged side chains of H20 and R75 are positioned ~3.0 Å from the carboxylate head group of decanoic acid, a distance consistent with electrostatic stabilization of ligand binding (Fig. 1). To evaluate if decanoic acid binding is dependent upon the electrostatic interactions, we determined the crystal structures of Rns–H20A, –R75A, and –H20A/R75A to 2.9, 2.35, and 2.4 Å resolution respectively. Overall, the structures of –H20A, –R75A, and –H20A/R75A were very similar to wild type. However, in each of the three variants, the binding pocket showed no electron density consistent with a bound fatty acid (Fig. 3). These results suggest that electrostatic interactions between the carboxylate head group of decanoic acid and the side chains of H20 and R75 are important for fatty acid binding.

**Fig 4:**
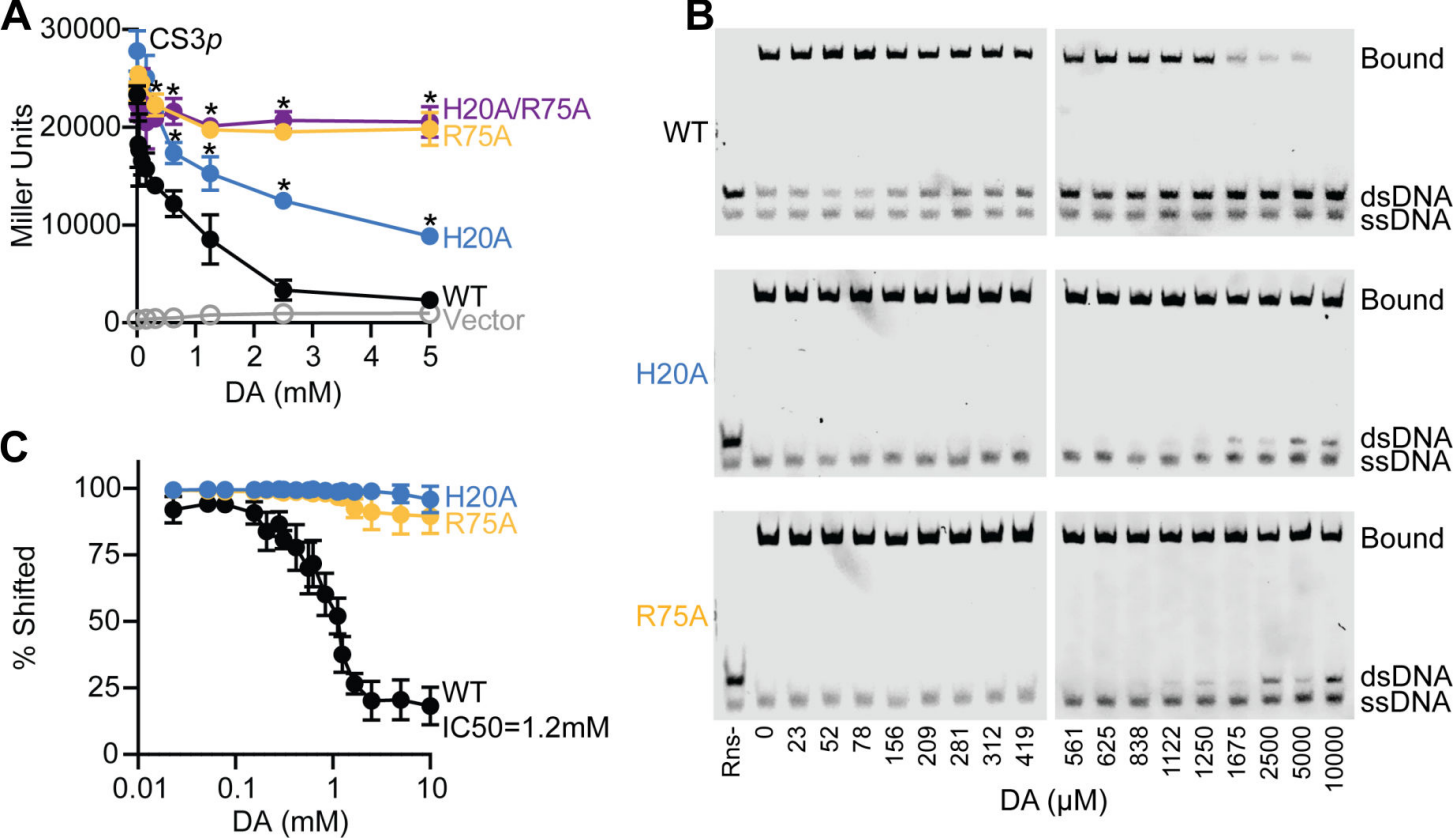
Decanoic acid inhibition is dependent on interactions between decanoic acid and Rns residues H20 and R75. (**A**) β-Galactosidase reporter assays of Rns-WT, H20A, R75A, and H20A/R75A in the presence of decanoic acid in 0.4% (vol/vol) DMSO. Data are shown as the mean response ±SD, *P < 0.05 by student’s *t*-test compared with WT activity. *n* = 3. (**B**) Representative EMSAs showing the effect of decanoic acid on 25 nM MBP-Rns binding to 5 nM DNA with a prototypical Rns binding site. (**C**) Quantification of *n* = 3 EMSAs using Licor Image Studio Lite. Data are shown as percent shifted (shifted band density divided by density of entire lane) ± SD. Decanoic acid inhibits DNA binding of Rns-WT with an IC50 of 1.2 mM and has no effect on DNA binding for Rns-H20A and R75A.

To further validate our crystallographic findings, we evaluated the activity and decanoic acid responsiveness of WT Rns and the three variants using a CS3p–Lac reporter strain. Expression of the ETEC CS3 pilus is Rns dependent, and DNaseI footprinting revealed a single Rns binding site immediately upstream of the promoter’s –35 hexamer (M. D. Bodero and G. P. Munson, unpublished data). In the absence of decanoic acid, the single and double alanine variants were active as determined by Rns-dependent expression of β-galactosidase in the CS3p–Lac reporter strain. Relative to the DMSO control, the activity of WT Rns decreased by 90% with 5 mM decanoic acid (Fig. 4). In contrast, the activity of Rns–R75A and –H20A/R75A was inhibited by 16% and 8%, respectively. Rns–H20A displayed an intermediate effect at 68% inhibition by decanoic acid.

In EMSAs, the IC50 for decanoic acid with WT Rns was determined to be 1.15 mM. The IC50 is estimated to exceed 10 mM decanoic acid for Rns–H20A and –R75A but could not be quantified due to the fatty acid’s inability to fully dislodge the variants from DNA (Fig. 4). The critical micelle concentration of decanoic acid also precluded titrations beyond ca. 15 mM. Interestingly, Rns–H20A appears to be less sensitive to decanoic acid by EMSA than Lac reporter assays. We attribute this inconsistency to intrinsic differences between the *in vitro* and *in situ* assays. These results indicate that the side chains of both H20 and R75 interact with the carboxylate head of decanoic acid and that fatty acid binding to the pocket abolishes the ability of Rns to bind DNA.

### Occlusion of the fatty acid binding pocket

**Fig 5:**
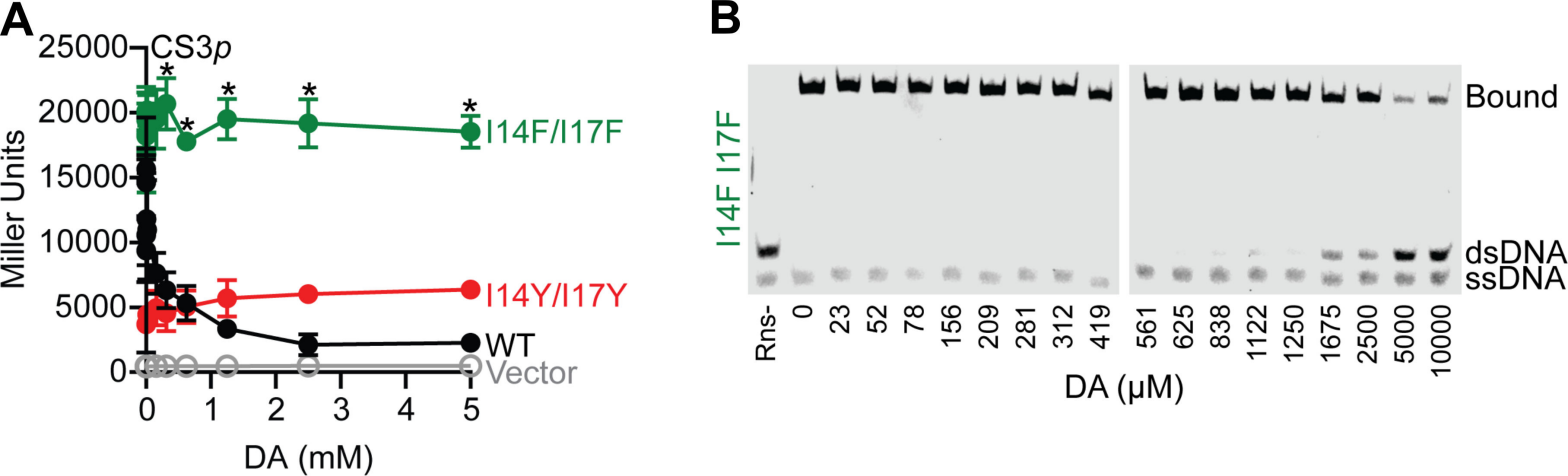
Rns-I14F/I17F is not inhibited by decanoic acid. (**A**) β-Galactosidase assays of Rns-WT, I14F/I17F, and I14Y/I17Y in the CS3p Lac reporter strain. Decanoic acid inhibits Rns-WT but does not have an effect on Rns-I14F/I17F or I14Y/I17Y. Data are given as the mean response ±SD, *P < 0.05 by Student’s *t*-test compared with WT activity. *n* = 3. (**B**) EMSA showing the effect of decanoic acid on 25 nM MBP-Rns-I14F/I17F binding to 5 nM DNA with a prototypical Rns binding site.

**Fig 6:**
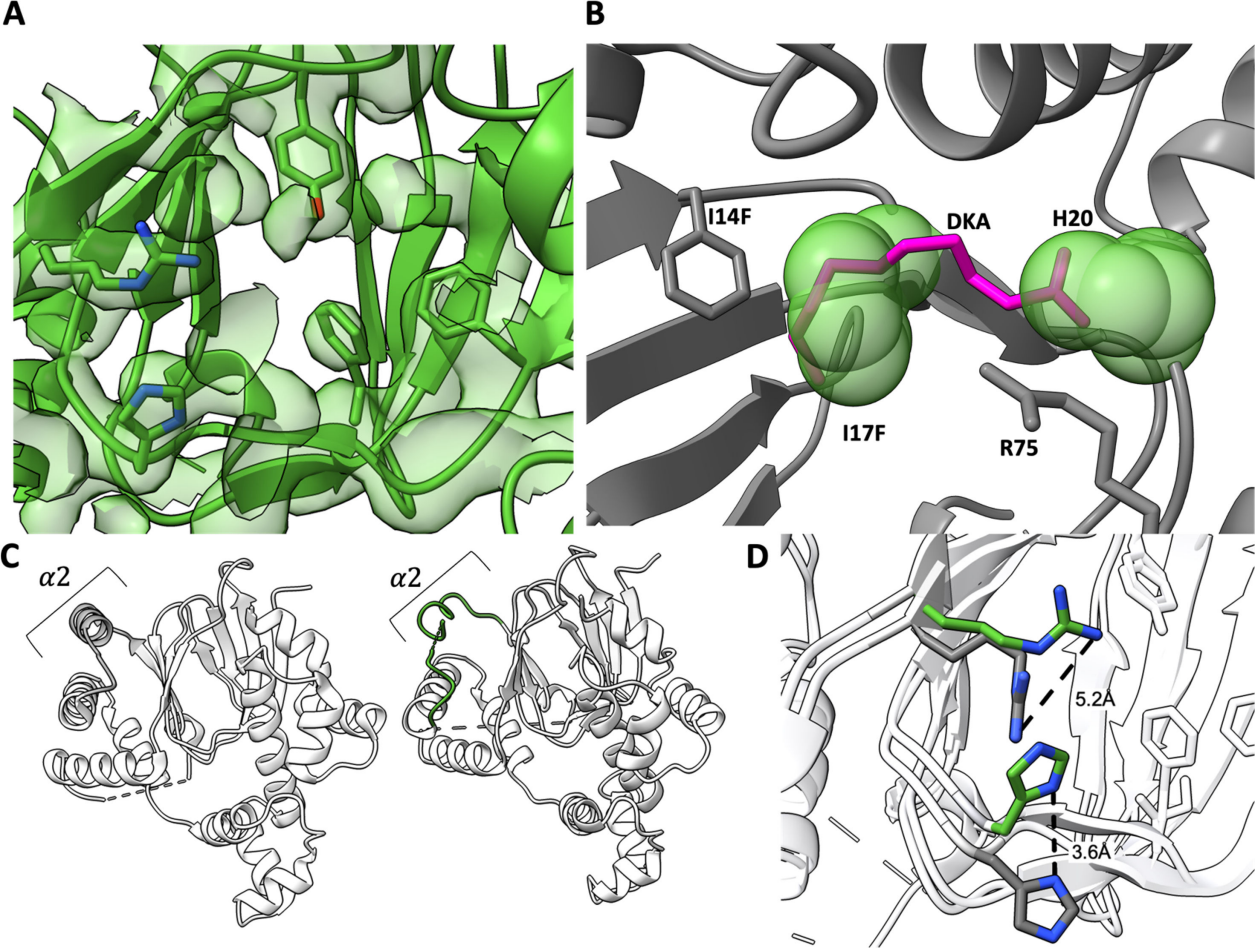
Analysis of the I14F/I17F pocket variant. (**A**) I14F/I17F (green, PDB ID: 9CA6) structure overlayed with corresponding electron density map shows no electron density consistent with bound decanoic acid. (**B**) Superposition of the position of decanoic acid from the wild type structure (pink) with the Rns-I14F/I17F structure. The Rns-I14F/I17F side chains that overlap with the decanoic acid are depicted in using space-filling spheres (green). (**C**) Helix α2 (gray in WT Rns) becomes disordered in the I14F/I17F (green) variant. (**D**) The side chain of R75 moves by 5.2 Å into the pocket of the I14F/I17F mutant when compared to WT, and the side chain of H20 moves 3.6 Å up and toward R75 in the I14F/I17F mutant.

To further investigate the structural requirements for ligand binding and response, we introduced mutations to sterically block ligand binding. Residues I14 and I17 in the hydrophobic region of the pocket were mutagenized to phenylalanine or tyrosine. Altering the residues to tyrosine made the protein insensitive to decanoic acid but also reduced basal activity, suggesting the protein was proteolytically unstable, and it was not analyzed further. As predicted, Rns–I14F/I17F was far less sensitive to decanoic acid inhibition than WT Rns; 25% vs. 75% max inhibitory activity (Fig. 5). As with the H20A and R75A variants, the I14F/I17F is also estimated to have an IC50 of >10 mM decanoic acid by EMSA. Although the I14Y/I17Y also appeared to be largely insensitive to decanoic acid in the CS3p–Lac reporter strain, it was not investigated further due to low overall activity relative to WT Rns and the I14F/I17F variant. To confirm that decanoic acid insensitivity was due to lack of fatty acid binding, we crystallized the I14F/I17F variant and solved its structure to 3.0 Å. As with the mutations that abolish electrostatic interactions with the carboxylate head group of decanoic acid, we observed no fatty acid-like electron density in the altered binding pocket (Fig. 6A). This is likely because the bulky side chain phenyl groups of F14 and F17 sterically block the position occupied by the aliphatic chain of decanoic acid in the WT structure (Fig. 6B). Unlike the electrostatic mutants, the structure of I14F/I17F differed from that of WT Rns. The side chains of both H20 and R75 moved into the pocket, with the imidazole ring of H20 occupying part of the space where the carboxylate head group of the decanoic acid ligand is in the WT structure (Fig. 6C).

### Overall structural analysis

**Fig 7:**
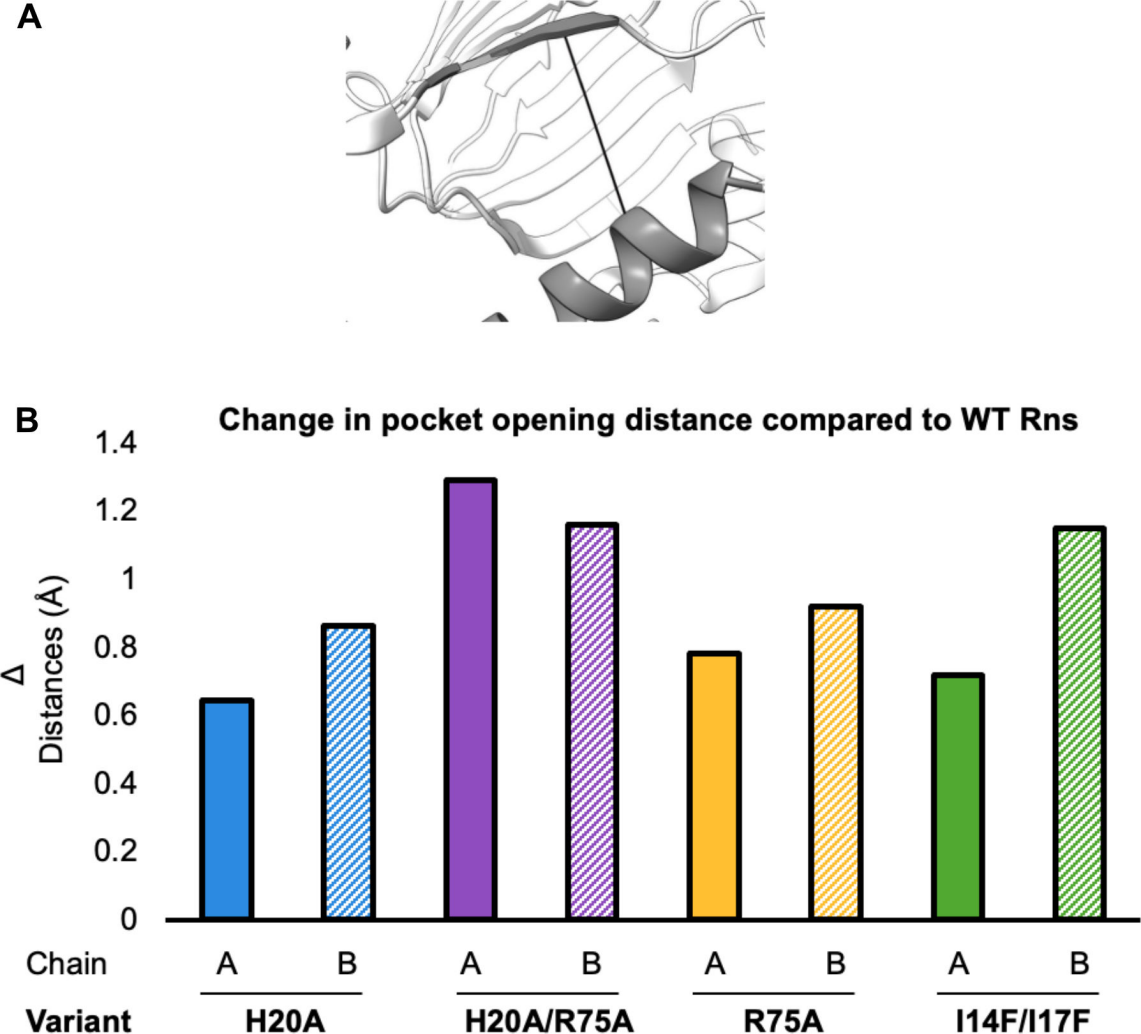
Global changes in the pocket of WT and variant Rns structures. (**A**) Widest point of pocket opening. All measurements are between the Cα of residues A73 and L219. (**B**) Change in distance compared with WT.

To determine if there were any structural differences between the previously published wild-type structure (36) and the altered proteins, we aligned the backbone atoms of the structures as well as compared their scaled B-factors. Overall, the structures and scaled B-factors are similar between all the structures. Aligning the backbone atoms between the wild-type and altered protein structures gave RMSDs of 0.574 to 1.17 Å^2^. Despite the structures being similar, there are two differences between the wild-type and altered proteins. First, the dimerization interface helix α2 in I14F/I17F is disordered, with part of the connection to helix α3 unresolved (Fig. 6D). The second difference is that all the mutant proteins show a more open pocket as measured from the long DBD helix α8 to β7 (residues 73 and 219). In the absence of ligand, the distance between the alpha-carbons of A73 and L219 in all mutants is increasing by ~1 Å (Fig. 7). Taken together, these results indicate that the altered proteins, which appear to be ligand-free, are more flexible than the wild-type protein.

To determine if there were differences in protein chain flexibility between the wild-type and altered proteins, the B-factors were scaled using inter-quartile scaling. Graphing the scaled Cα B-factors for the wild-type and altered proteins showed they followed a similar pattern across the structures (Fig. S2), with one notable exception. The Rns-I14F/I17F structure has more disorder in the first DNA binding helix compared with the other structures. This suggests changes in flexibility are reflected in the structures as seen above and not necessarily reflected in the B-factors.

### Specificity/promiscuity of the fatty acid binding pocket

**Fig 8:**
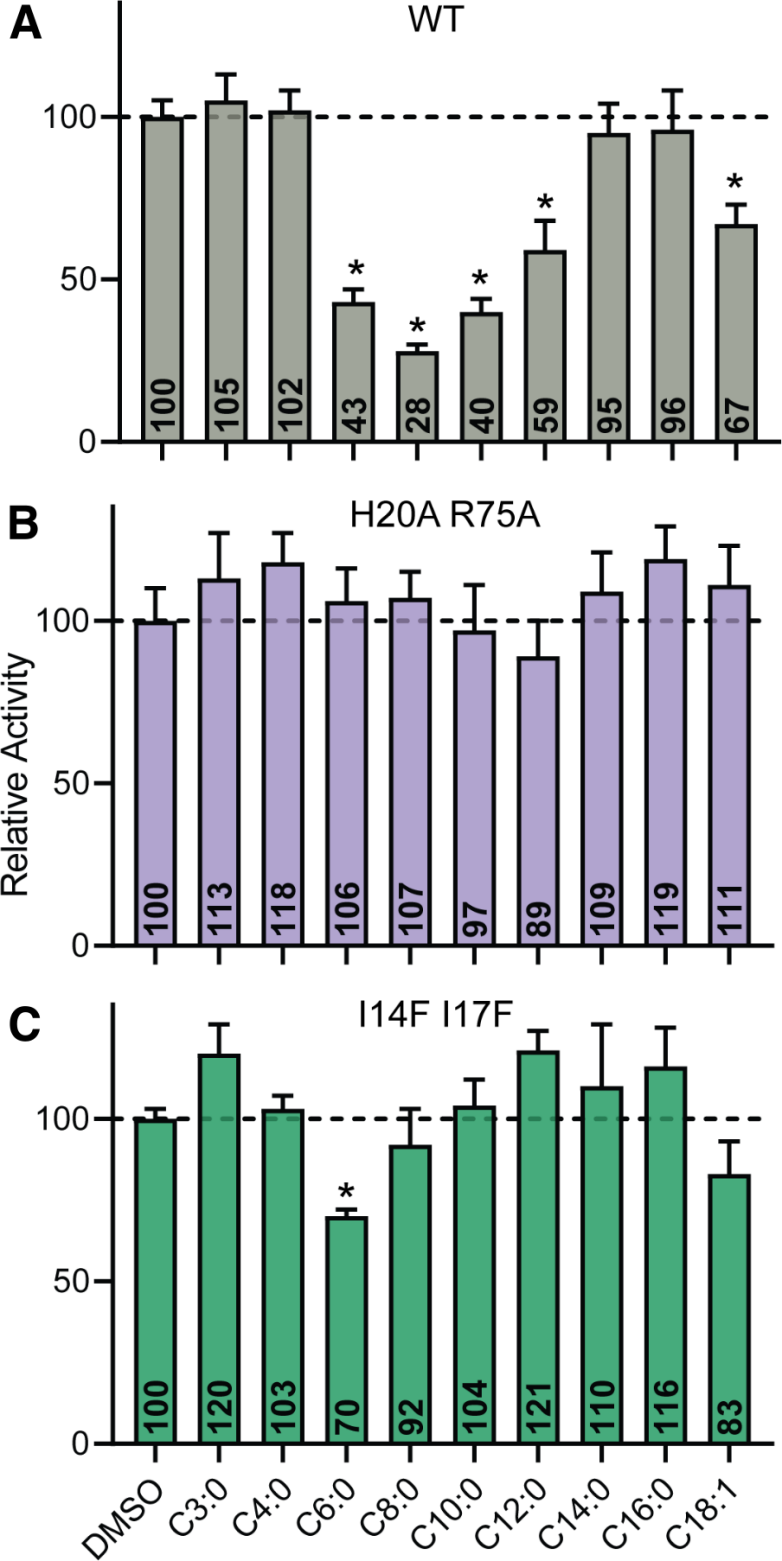
The specificity of the Rns fatty acid binding pocket. β-Galactosidase assays of (**A**) Rns-WT, (**B**) H20A/R75A, and (**C**) I14F/I17F expressed in CS3p reporters in the presence of 3 mM (0.4% [vol/vol] final DMSO) of saturated fatty acids C3-C16 or monounsaturated fatty acid C18:1. Data are given as mean relative activity (activity normalized to DMSO × 100) ± SD, *n* = 3. *P < 0.002 to DMSO activity via Student’s *t*-test.

Although Rns was initially crystallized with decanoic acid, we considered the possibility that other fatty acids may also occupy the pocket and inhibit the activity of Rns. Indeed, hexanoic acid (C6), octanoic acid (C8), and dodecanoic acid (C12) saturated fatty acids significantly inhibited the activity of Rns in our β-galactosidase assay (Fig. 8A). We also observed inhibition by oleic acid (C18:1), an unsaturated fatty acid. The Rns-H20A/R75A variant was found to be insensitive to the same fatty acids (Fig. 8B). These results suggest that the carboxylate head groups of each are coordinated by electrostatic interactions with the side chains of H20 and R75, as we observed for decanoic acid. With the exception of hexanoic acid, occlusion of the binding pocket in Rns-I14F/I17F also abolished inhibition by the fatty acids (Fig. 8C). Although the I14F/I17F mutation may not fully prevent binding of all fatty acids, this mutation did decrease the effectiveness of inhibition relative to WT Rns.

## DISCUSSION

There is a growing body of evidence that many AraC-virulence regulators (AraC-VR) from enteric bacteria are inhibited by fatty acid ligands. Since it was found that monounsaturated long-chain fatty binding to *V. cholerae* ToxT inhibits virulence gene transcription by disrupting DNA binding (29, 32), others have shown that fatty acids similarly inhibit virulence gene expression in *Salmonella* (HilD, HilC, and RtsA) (30, 33). We recently showed that fatty acid binding to Rns from ETEC also inhibits virulence gene expression and identified a number of additional AraC-family members that we hypothesized, based on predicted structural similarities, could be regulated in a similar manner, including FapR (ETEC), PerA (EPEC), HilC (*Salmonella typhi*), VirF (*Shigella flexneri*), and VirF (*Yersinia enterocolitica*). Supporting this hypothesis, it was recently shown that VirF from *S. flexneri* is indeed regulated by medium-chain saturated and long-chain unsaturated fatty acids (34). The two structures of full-length AraC-VRs with ligand bound, ToxT and Rns, inform on the molecular mechanisms of fatty acid inhibition of this group of AraC-VRs; however, a thorough exploration of the binding pocket has been lacking. In this study, we comprehensively investigated the roles of binding pocket residues in ligand interactions and regulating Rns activity.

This study clearly demonstrates that residues making up the binding pocket are important for ligand binding and subsequent disruption of Rns-DNA interactions. Altering the amino acids which interact with the carboxylic head group reduced the ability of decanoic acid to inhibit DNA binding. Furthermore, structural studies and EMSAs support our hypothesis that the altered proteins are not able to bind ligand. Interestingly, the Rns H20A protein showed an intermediate phenotype in the β-galactosidase assay, differing from the EMSA which showed almost complete inhibition out to 10 mM decanoic acid. This discrepancy could be the result of the different assay conditions. However, R75 turned out to be the most important charged residue in the pocket, as altering it completely abolished ligand-dependent inhibition as well as the ability of Rns to bind decanoic acid.

In addition to removing the charge interactions, we also altered pocket side chains to block decanoic acid binding to determine if filling the pocket with larger side chains would block decanoic acid binding, or if the pocket was plastic enough to still accommodate the fatty acid. Altering I14 and I17 to introduce bulky phenylalanine side chains blocked decanoic acid binding, resulting in Rns being insensitive to ligand in both β-galactosidase and EMSA assays. These results suggest one of two things: either that it is important for the ligand to fill the binding pocket, or that binding of the ligand tail correctly positions the carboxylate head group to achieve inhibition.

Overall, the structures of the altered Rns proteins are similar to the wild type structure. However, subtle differences suggest that ligand-free wild type protein is more flexible than the ligand-bound protein. The ligand binding pocket is slightly more open in the apo altered proteins than the ligand-bound wild type. Also, the Rns I14F/I17F structure showed considerable differences in the α2 region, where the helix became unstructured. These results suggest that apo wild-type Rns has increased flexibility when compared with ligand-bound Rns; however, further experiments are required to verify these protein dynamics.

Fatty acid inhibition of DNA binding by Rns, ToxT, and other AraC-VRs is a significant departure from the AraC paradigm in which ligand binding alters the occupancy of binding sites but not DNA binding *per se* (26). Based on our studies to date, we hypothesize that ligand binding rigidifies the protein, making it unable to bind to DNA. Support for this hypothesis can be found in the ToxT structure, where the DNA binding domain is more disordered in the apo state than when ligand is bound (42, 43). However, as above, further experimentation examining protein dynamics is required to answer this question.

Future work will investigate the mechanism by which ligand binding regulates Rns DNA binding activity, as well as to determine how dimerization affects Rns activation. We also plan to directly measure the physical properties of fatty acid binding to Rns. Such information will nicely complement the results of this study that have identified amino acid side chains in the pocket that are required for Rns to be inhibited by fatty acids. In particular, the fatty acid must be accommodated by the pocket, and the carboxylic head group must bind to R75 for Rns to be inhibited. More generally, this study demonstrates that many AraC-VRs utilize the same regulatory mechanism in which fatty acids must bind in the pocket to inhibit DNA binding.

## MATERIALS AND METHODS

### Plasmid site-directed mutagenesis

Plasmid pGPMRns-Myc was PCR amplified with primers 1565/1566, 1567/1568, and 2410/2411 to generate *rns* H20A, R75A, and I14F/I17F, respectively. The PCR products were circularized with NEB HiFi to construct pGPMRns-Myc H20A, R75A, H20A/R75A, and I14F/I17F for use in β-galactosidase assays.

Rns expression plasmids for protein expression were constructed by PCR amplification of pCDB24-Rns (36) with point mutation primers designed using SnapGene, followed by ligation with the KLD kit (NEB). The resulting plasmids were transformed into DH5α’s, plated onto LB agar with 100 µg/mL carbenicillin and incubated overnight at 37°C. Selected colonies were used to inoculate ZYP-0.8G, 100 µg/mL carbenicillin and grown overnight at 37°C. Plasmids were isolated using the Qiagen miniprep kit and the mutations were verified by sequencing. Sequence-verified plasmids were then transformed into BL21 DE3 cells (NEB). Oligonucleotides used in this study are listed in Table S1.

### MBP-Rns expression plasmids

Codon-optimized Rns was amplified with primers 2469/2497 from pCDB24-Rns (WT) (36), pJDT037 (H20A), pJDT011 (R75A), pJDT039 (H20A/R75A), or pJDT104 (I14F/I17F). The PCR products and pMalC2 were digested with BamHI and HindIII, then ligated, resulting in pMBPRnsOPT2 WT, H20A, R75A, H20A/R75A, and I14F/I17F, respectively. All plasmid sequences were confirmed via Sanger sequencing. Plasmids used in this study are listed in Table S2.

### β-Galactosidase assays

Lac reporter strain GPM1072 was transformed with pGPMRns-Myc plasmids (WT, H20A, R75A, H20A/R75A, and I14F/I17F) or vector pTags2. All strains were grown aerobically at 37°C to stationary phase in LB medium with 100 µg/mL ampicillin with or without decanoic acid in 0.4% (vol/vol) DMSO. β-Galactosidase activity was assayed as previously described (44). All strains used in this study are listed in Table S3.

### Rns variant crystallization

The Rns variants were cultured and purified as described in (36) with the following changes (36). Lysis was performed with sonication on ice at 50% power for 7 min, cycling on for 30 sec and off for 30 sec. The remainder of the purification was the same.

Initial crystal trays were set up using 96-well evolution plates. The condition described in reference 36 was used as a starting point. While other matrix screens from Qiagen or Hampton were tried, the original condition was found to produce the most consistent crystals of the Rns variants. Interestingly, a round of screening with the additive screen revealed that DMSO improved crystal morphology, for a final crystal condition of 1:2 DMSO to final protein and condition (0.1 M succinic acid, 14% PEG 3350, 0.03 M glycyl-glycyl-glycine) volume. The cryogenic condition used for all crystals was 1:1 glycerol and reservoir solution resulting in 50% glycerol cryo-conditions

### Rns variant data collection, structural determination, and analysis

Diffraction data of the Rns variants were collected at NSLS2 using either the AMX or FMX beamlines. Initial data reduction was performed with XDS (36, 45). Molecular replacement was performed using PHASER with the wild-type Rns PDB:6XIU as the model (36). Automated refinement was carried out in PHENIX, and manual model building was done using COOT (46, 47). Final models were deposited into the PDB (48). See Table S4 for data and model statistics.

ChimeraX was used for structural analysis and visualization (49). To analyze the structures, they were first aligned in Matchmaker (50) using the long helix in the DNA binding domain (N204-E223) as a fiducial marker. The opening between the N- and C-terminal domain was measured by selecting the Cα of the amino acids 73 and 219 and then using ChimeraX to obtain the distances. To obtain the overall Cα RMSD of the structures, the Cα atoms were selected and then aligned in Matchmaker. Each chain was aligned separately.

To compare the B-factors of the different structures, it was first necessary to scale the B-factors to a common median (51). To scale the B-factors of the PDBs of the structures, only the lines describing protein atom positions were left, i.e., all water and heteroatoms were removed. The data were then imported into STATA (STATACorp) so all the PDBs were in one data set. Scaling was carried out using a robust scaling method, so the median B-factor was set to 0, and the interquartile range was set to one for each chain. The results were graphed using STATA.

### Expression and purification of MBP-Rns

Maltose binding protein (MBP) fused to the amino terminus of Rns was expressed and purified with a protocol modified from reference 12. BL21 cells were transformed with pMBPRnsOpt2 WT, H20A, R75A, or I14F/I17F and grown aerobically at 37°C in LB medium with 200 µg/mL ampicillin and 2 mM MgCl_2_. After reaching mid-log phase, cultures were transferred to a 30°C shaking water bath, and expression of MBP-Rns was induced by the addition of 1 mM IPTG. After overnight induction, cells were harvested at 6,000 × *g* for 10 min at 4°C and concentrated 100-fold in ice-cold lysis buffer A (20 mM Tris-Cl [pH 7.6], 200 mM NaCl, 1 mM EDTA). Cells were lysed by two to three passages through a French press. Insoluble material was removed by centrifugation at 40,000 × *g* for 40 min at 4°C. The supernatant was loaded onto a 1 mL MBPTrap HP column (Cytiva) and eluted from the column with buffer A with 10 mM maltose. Fractions containing MBP-Rns were loaded onto a HiPrep Heparin FF 16/10 column (Cytiva) in heparin buffer (20 mM Tris-Cl pH 8, 150 mM NaCl) and eluted via a step gradient with heparin buffer with 1 M NaCl. Fractions containing MBP-Rns were loaded onto a Pall Centrifuge filter for buffer exchange to storage buffer (10 mM Tris-Cl [pH 7.4], 280 mM NaCl, 1 mM EDTA, 10 mM β-mercaptoethanol, 25% [vol/vol] glycerol) and stored at −80°C. Protein concentration was determined with the Bio-Rad Bradford assay relative to a BSA standard curve.

### Electrophoretic mobility shift assays

Oligonucleotides 2107/2108 that encode a prototypical Rns binding site (Table S1: 2107/2108) that is 5′ cyanine 5.5 tagged on both strands were duplexed by heating at 65°C for 10 min followed by slow cooling to room temperature over greater than 1 h. Then, 30 nM purified MBP-Rns was incubated with decanoic acid (final concentration 2% [vol/vol] DMSO) in binding buffer [10 mM Tris-Cl (pH 7.4), 50 mM KCl, 1 mM DTT, 1 ng/µL poly(dI-dC), and 100 µg/mL BSA] for 1 h at room temperature. Subsequently, 5 nM duplex probe was added to each reaction followed by an additional incubation at room temperature for 10 mins. Glycerol was added to a final concentration of 6.5% (vol/vol) prior to separation on 5% native polyacrylamide gels. Gels were analyzed with the Odyssey FC Imaging System (LI-COR Biosciences).

## Data Availability

The Rns variant structures were deposited into the PDB; RnsH20A (PDBID: 9CA5), RnsR75A (PDBID: 8VRQ), RnsH20A/R75A (PDBID: 8VST), and RnsI14F/I17F (PDBID: 9CA6).

## SUPPLEMENTAL MATERIAL

Supplemental material msphere.00115-25-s0001.docx Supplemental tables and figures.

ASM does not own the copyrights to Supplemental Material that may be linked to, or accessed through, an article. The authors have granted ASM a non-exclusive, world-wide license to publish the Supplemental Material files. Please contact the corresponding author directly for reuse.
