# Supplementary material for: Characterization of the ligand binding pocket of the virulence regulator Rns, a member of the AraC/XylS family of transcription factors
Source: mSphere. 2025 Jul 24;10(8):e00115-25. doi: 10.1128/msphere.00115-25 (PMC12379584; doi:10.1128/msphere.00115-25)
Supplement: Supplemental material — Supplemental tables and figures. [file msphere.00115-25-s0001.docx]

**Supplemental Information**

| **Table S1: Primers used in this study** | |
| --- | --- |
| **Name** | **Sequence** |
| 1565 | ATGATTgcgAAATACACTGTATTATATACATC |
| 1566 | CAGTGTATTTcgcAATCATAATATTATTAATTTTTATTG |
| 1567 | GCATTCgcgTTGAACGGAGATATGCTAAG |
| 1568 | CGTTCAAcgcGAATGCAACATACGGC |
| 2107 | [Cy5.5]-TAGATGGAAT**GTGTTATTTTTTTATC**AAAATTATATTTGT |
| 2108 | [Cy5.5]-ACAAATATAATTTT**GATAAAAAAATAACAC**ATTCCATCTA |
| 2410 | AAAAtttAATAATtttATGATTCATAAATACACTGTATTAT |
| 2411 | TCATaaaATTATTaaaTTTTATTGTTTCTTTTTCTTCAG |
| 2496 | gcggatccGATTTTAAATACACGGAAGAAAAAGAG |
| 2497 | gcgaagcttTTAACCACCTTTGAAGTACGT |
| Rns-R75A-F | CGTCGCCTTCgcaTTGAATGGAGAC |
| Rns-R75A-R | TATGGTTTCTCGCTTAAG |
| Rns-H20A-F-New | CATTATGATCgctAAGTACACCGTGC |
| Rns-H20A-R | TTGTTGATTTTGATAGTCTCTTTTTC |
| Rns-I14F/I17F-F | AACtttATGATCCATAAGTACACC |
| Rns-I14F/I17F-R | GTTgaaTTTGATAGTCTCTTTTTCTTCC |

*Lowercase indicates primer-template mismatch for site-directed mutagenesis or the addition of restriction endonuclease sites. Bold denotes Rns binding site.*

| **Table S2: Plasmids used in this study** | | | |
| --- | --- | --- | --- |
| **Name** | **Description** | **Marker** | **Reference** |
| pGPMRns-Myc | Rns expressed from *lacp* | *bla* | [1] |
| pGPMRns-Myc H20A | Rns H20A expressed from lacp | *bla* | This study |
| pGPMRns-Myc R75A | Rns R75A expressed from lacp | *bla* | This study |
| pGPMRns-Myc H20A/R75A | Rns H20A/R75A expressed from lacp | *bla* | This study |
| pGPMRns-Myc I14F/I17F | Rns I14F/I17F expressed from lacp | *bla* | This study |
| pTags2 | Cloning vector | *bla* | [2] |
| pMal-C2 | MBP cloning vector |  | New England Biolabs |
| pCDB24-Rns | 10xHis-SUMO(SMT)-Rns |  | [1] |
| pJDT037 | SMT-Rns-H20A |  | This study |
| pJDT011 | SMT-Rns-R75A |  | This study |
| pJDT039 | SMT-Rns-H20A/R75A |  | This study |
| pJDT104 | SMT-Rns-I14F/I17F |  | This study |
| pMBPRnsOpt2 | IPTG inducible codon optimized MBP-Rns from tacp |  | This study |

| **Table S3: Strains used in this study** | | |
| --- | --- | --- |
| **Strain** | **Characteristics** | **Reference** |
| GPM1072 | *E. coli* K-12 MC4100 *attB*_HK022_::pCS3Lac1 | [1] |
| GPM1080 | *E. coli* K-12 MC4100 *attB*_HK022_::pNlpALac1 | [3] |
| JT0040 | E. coli fhuA2 [lon] ompT gal (λ DE3) [dcm] ∆hsdS (RnsR75A) | This study |
| JT064 | E. coli fhuA2 [lon] ompT gal (λ DE3) [dcm] ∆hsdS (RnsH20A) | This study |
| JT066 | E. coli fhuA2 [lon] ompT gal (λ DE3) [dcm] ∆hsdS (RnsH20A/R75A) | This study |
| JT175 | E. coli fhuA2 [lon] ompT gal (λ DE3) [dcm] ∆hsdS (RnsI14/I17F) | This study |

Table References:

1. Midgett, C.R., Talbot, K.M., Day, J.L. et al. Structure of the master regulator Rns reveals an inhibitor of enterotoxigenic Escherichia coli virulence regulons. Sci Rep 11, 15663 (2021).
2. Munson, George (2020): Maps and full sequences of bacterial expression plasmids pTags2, pTags2-Hyg1, and pTags2-Zeo1. The NIH Figshare Archive. Figure. <https://doi.org/10.35092/yhjc.12469133.v1>
3. Bodero, M.D., Pilonieta C.M., Munson G.P., Repression of the Inner Membrane Lipoprotein NlpA by Rns in Enterotoxigenic Escherichia coli. J Bacteriol, 189. (2006)

| **Table S4: Crystallographic Statistics for all mutants** | | | | | | | |  |
| --- | --- | --- | --- | --- | --- | --- | --- | --- |
|  | **RnsR75A**  **(8VRQ)** |  | **Rns H20A/R75A**  **(8VST)** |  | **Rns I14FI17F**  **(9CA6)** |  | **H20A**  **(9CA5)** | |
| **Resolution range** | 29.22 - 2.4  (2.486 - 2.4) |  | 48.65 - 2.4  (2.486 - 2.4) |  | 29.2 - 2.995  (3.1 - 3.0) |  | 46.1 - 2.9  (3 - 2.9) | |
| **Space group** | P 21 21 21 |  | P 21 21 21 |  | P 21 21 21 |  | P 21 21 21 | |
| **Unit cell** | 47.73 96.97 136.59  90 90 90 |  | 47.65 97.29 136.94  90 90 90 |  | 47.81 96.68 138.15  90 90 90 |  | 47.73 97.93 136.69  90 90 90 | |
| **Total reflections** | 161945 (15846) |  | 170086 (17399) |  | 68908 (6923) |  | 90422 (9910) | |
| **Unique reflections** | 25501 (2497) |  | 25620 (2499) |  | 24421 (2403) |  | 25426 (2759) | |
| **Multiplicity** | 6.4 (6.3) |  | 6.6 (7.0) |  | 2.8 (2.9) |  | 3.6 (3.6) | |
| **Completeness (%)** | 99.55 (99.64) |  | 99.70 (99.84) |  | 98.98 (98.91) |  | 92.35 (99.18) | |
| **Mean I/sigma(I)** | 7.03 (0.74) |  | 6.49 (0.78) |  | 2.32 (0.44) |  | 3.08 (0.38) | |
| **Wilson B-factor** | 63.73 |  | 60.41 |  | 77.99 |  | 87.44 | |
| **R-merge** | 0.1517 (2.973) |  | 0.1837 (2.423) |  | 0.3782 (2.264) |  | 0.2496 (3.402) | |
| **R-meas** | 0.1659 (3.239) |  | 0.1998 (2.617) |  | 0.4687 (2.746) |  | 0.295 (4.008) | |
| **R-pim** | 0.06641 (1.273) |  | 0.07736 (0.9801) |  | 0.2723 (1.533) |  | 0.1558 (2.099) | |
| **CC1/2** | 0.996 (0.395) |  | 0.995 (0.383) |  | 0.928 (0.146) |  | 0.986 (0.163) | |
| **CC*** | 0.999 (0.752) |  | 0.999 (0.744) |  | 0.981 (0.506) |  | 0.996 (0.529) | |
| **Reflections used in refinement** | 25457 (2489) |  | 25595 (2497) |  | 13327 (1272) |  | 13705 (1448) | |
| **Reflections used for R-free** | 1997 (196) |  | 2000 (195) |  | 1333 (128) |  | 1372 (146) | |
| **R-work** | 0.2383 (0.3223) |  | 0.2467 (0.3711) |  | 0.3488 (0.4226) |  | 0.2641 (0.4099) | |
| **R-free** | 0.2869 (0.3469) |  | 0.2971(0.3853) |  | 0.3678 (0.4689) |  | 0.3149 (0.4560) | |
| **Number of non-hydrogen atoms** | 4151 |  | 4026 |  | 3939 |  | 4025 | |
| **macromolecules** | 4137 |  | 4011 |  | 3939 |  | 4025 | |
| **Protein residues** | 508 |  | 493 |  | 481 |  | 493 | |
| **RMS(bonds)** | 0.01 |  | 0.01 |  | 0.009 |  | 0.006 | |
| **RMS(angles)** | 1.13 |  | 1.18 |  | 1.09 |  | 0.75 | |
| **Ramachandran favored (%)** | 97.6 |  | 98.76 |  | 96.79 |  | 96.91 | |
| **Ramachandran allowed (%)** | 2.4 |  | 1.24 |  | 3.21 |  | 2.89 | |
| **Ramachandran outliers (%)** | 0 |  | 0 |  | 0 |  | 0.21 | |
| **Rotamer outliers (%)** | 1.27 |  | 2.62 |  | 2 |  | 1.3 | |
| **Clashscore** | 8.72 |  | 10.56 |  | 8.91 |  | 8.31 | |
| **Average B-factor** | 70.89 |  | 69.54 |  | 82.21 |  | 101.08 | |


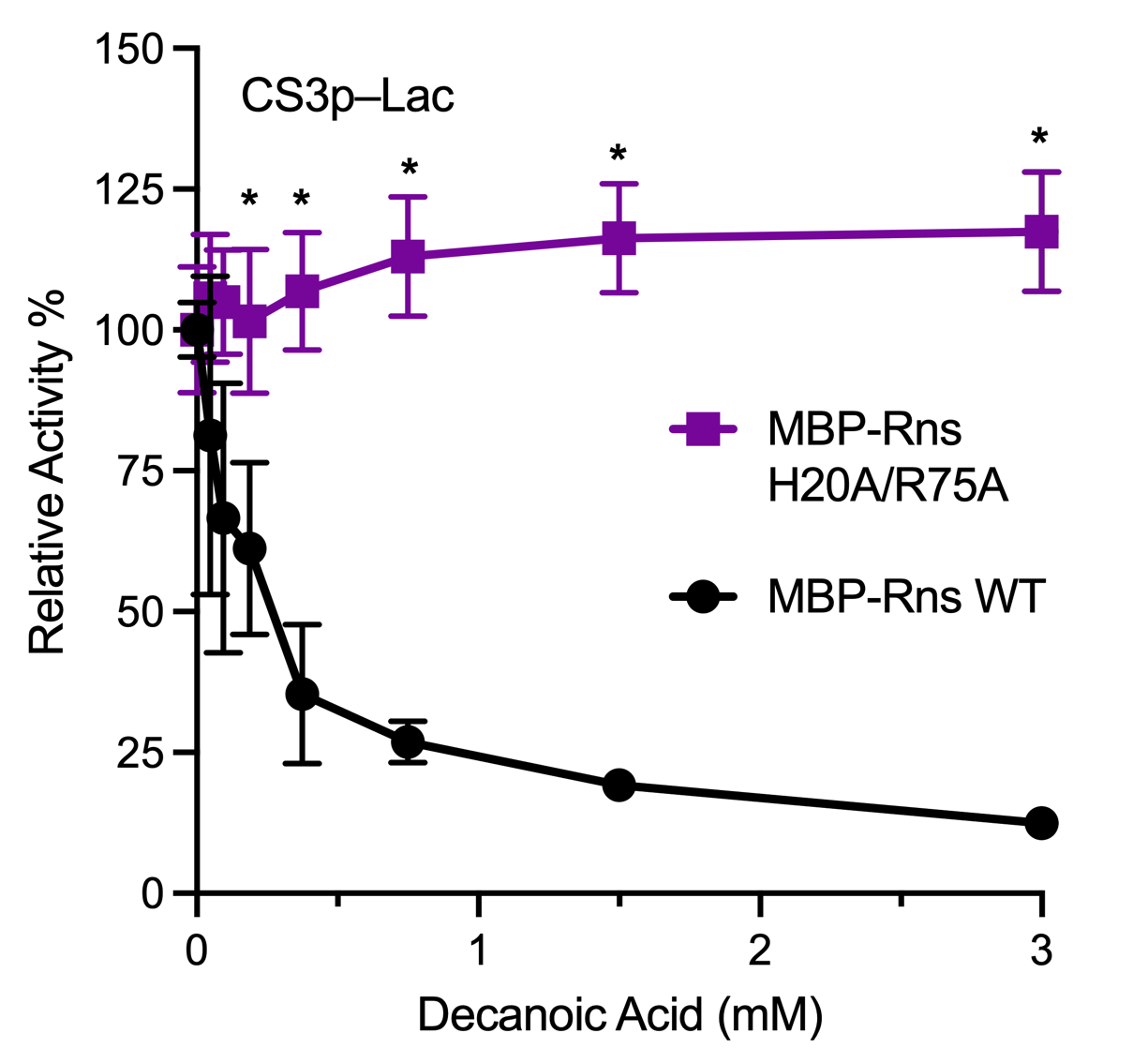


**Figure S1**. **The MBP solubility tag does not interfere with decanoic acid inhibition of Rns.** MBP-Rns WT and H20A/R75A activation of the CS3p-Lac reporter in the presence of decanoic acid in 0.4% (v/v) DMSO. Mean β-galactosidase Miller units were normalized to DMSO only controls. **P* < 0.05 by Student’s t-test compared to WT activity, *n* = 3.


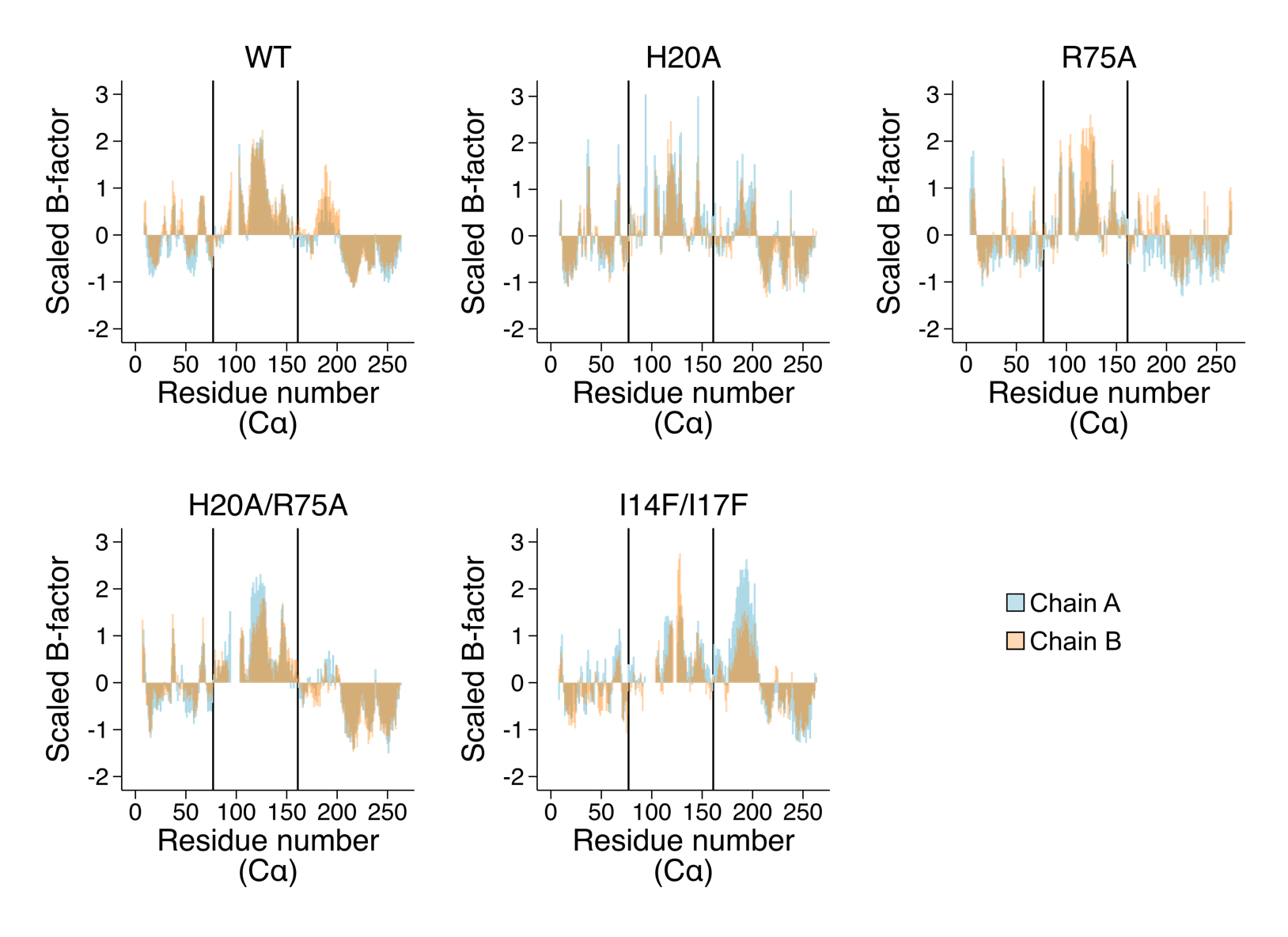
**Figure S2. Scaled B-factors of Rns wildtype and altered protein structures.** Graphs of the scaled B-factors from the different structures are shown. There are two lines drawn at residue 77 and residue 161 on each plot, separating them into three regions. From left to right the first region is most of the binding pocket, followed by the dimerization helices, and finally the DNA binding domain.
